# Systems approaches to support action on physical activity

**DOI:** 10.2471/BLT.20.250936

**Published:** 2020-01-31

**Authors:** Harry Rutter, Nick Cavill, Adrian Bauman, Fiona Bull

**Affiliations:** a Department of Social and Policy Sciences, University of Bath, Claverton Down, Bath BA2 7AY, England.; b Centre for Exercise, Nutrition and Health Sciences, University of Bristol, Bristol, England.; c School of Public Health, Sydney University, Sydney, Australia.; d Department of Health Promotion, World Health Organization, Geneva, Switzerland.

We note the response from Nuzzo & Steele^1^ to our perspective published in the February 2019 issue of this journal.^2^ In that article we presented an initial physical activity system map.^2^ However, we refute their criticisms of our paper, which appear both to represent a misreading of it and to demonstrate an erroneous conflation of systems science with systems thinking. Nuzzo & Steele create a false equivalence between conceptual maps designed to aid the development and planning of policy and practice, and epidemiologically formal maps that are designed to describe detailed specifics of causation.

The initial physical activity system map we included in our paper was explicitly intended to support the identification of potential mechanisms for influencing the determinants of physical activity and to help with communicating the need for wide-ranging actions across multiple sectors and domains. We clearly stated that “the map does not aim to be a formal causal loop diagram with balancing and reinforcing loops, nor does it attempt to quantify the nature of the relations between factors”. Nuzzo & Steele claim that “the purpose of systems maps should be to reflect causality… to depict relationships between variables which, if acted upon, cause predictable changes in physical activity.”^1^ Maps that do this can unquestionably be useful, but it may take many years and extensive research programmes to develop the required evidence to underpin them, and they serve a very different purpose from ours. Thus, to conflate the two is to miss one of the core points that we were making.

We strongly support systems science approaches, including causal inference and modelling, where appropriate. But these techniques are not always needed and may not even be feasible. As Nuzzo & Steele themselves point out, a formal causal map for physical activity “would require an updated systematic review of the literature on all possible correlates and determinant of physical activity, as well as associated confounders and moderators, followed by careful application of the rules of directed acyclic graphs to produce an accurate and informative map.”^1^ We would strongly argue that this approach is not necessary to identify the most important plausible and modifiable determinants of physical activity to support the adoption of evidence-informed policy and practice pathways to underpin strategic and comprehensive planning approaches.^3^

A core challenge inherent in a complex systems approach to physical activity promotion is that the existing evidence base has largely been generated on the basis of linear models of cause and effect relating to discrete interventions, longitudinal observations or natural experiments. Complex systems, however, display characteristics, such as feedback, adaptation and nonlinearity, and are highly dependent on context.^4^ These characteristics make it difficult, and in some cases impossible, to quantify the contributions of the multiple determinants that may be necessary for the effective promotion of physical activity, but none of which is sufficient on its own to achieve it. Restricting the factors in a map only to those for which robust causal relations are known would thus generate a limited and skewed set of factors and relations, which would be unlikely to achieve population change. This would risk perpetuating the policy failures that have led to a lack of substantive improvements in levels of physical activity across much of the world.^5^

We face a global epidemic of noncommunicable diseases, and physical activity promotion provides an important opportunity to improve health and reduce inequalities.^6^ There is unquestionably a place for rigorous attempts to ascertain ever more detailed aspects of the causal relations between the drivers of activity beyond reasonable doubt. However, our paper describes a different kind of approach that we believe can help with the urgent task of identifying effective ways to engage policy-makers, practitioners, other stakeholders and the public in creating supportive environments that promote sustained physical activity across the life course. We need to place a much higher priority on the pragmatic kind of process we argue for in our paper, an approach that complements, but has a quite different purpose from the more epidemiologically rigorous causal research favoured by Nuzzo & Steele. We fully acknowledge that there is no gold standard for such a map – as we said in our paper, a different group would produce a different map, and to date, little empirical evidence exists on the value of these maps. But existing approaches have failed to reverse the crisis of inactivity and it is time to try, and of course evaluate, new approaches. The approach we describe should not be considered as an attempt to depict the supposed specifics of a fixed reality, but as a tool to aid new ways of using systems thinking to help drive effective action.
